# Effect of Laser Scanning Parameters on Surface Morphology and Topography of Glass Solder-Coated Zirconia Substrate

**DOI:** 10.3390/jfb16090324

**Published:** 2025-09-03

**Authors:** Fiona Hartung, Christian Moss, Hermann Seitz, Georg Schnell

**Affiliations:** 1 Chair of Microfluidics, Faculty of Mechanical Engineering and Marine Technology, University of Rostock, Justus-von-Liebig Weg 6, 18059 Rostock, Germany; hermann.seitz@uni-rostock.de; 2 Moss Laboratorium für Zahn- und Implantattechnik GmbH, Sachsenfeld 3-5, 20097 Hamburg, Germany; christian.moss@dentallabor-moss.de; 3 Department Life, Light & Matter, University of Rostock, Albert-Einstein-Str. 25, 18059 Rostock, Germany

**Keywords:** femtosecond laser, laser-structuring, glass, antibacterial surface, osseointegration, dental implant, biofilm

## Abstract

Surface roughness and morphology, along with surface chemistry, are key features for improving ingrowth behavior and combating peri-implantitis after the insertion of dental implants. Using femtosecond laser texturing, this study aims to control both morphological and topographical surface properties of a glass solder coating on a zirconia substrate for dental applications. Experiments with varying laser and scanning parameters on the upper glass solder layer show the occurrence of two different surface morphologies. On the one hand, periodic wave-like structures are generated at relatively low pulse energy, with a high scanning pulse overlap of 80 to 90% and a scanning line overlap of 50%. On the other hand, a cauliflower-like structure can be observed at high pulse energies and a line overlap of up to 90%. Both surface morphologies represent a potential way to modify the glass solder surface to customize hard- and soft-tissue ingrowth, while realizing anti-adhesive properties for pathogenic bacteria in dental applications.

## 1. Introduction

**Figure 1:**
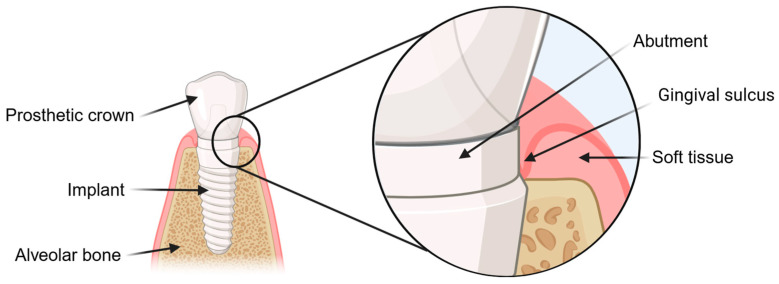
Components of a dental implant and surrounding dental structures. The three components of a dental implant are the implant, abutment and prosthetic crown. The attachment of soft tissue and osseointegration are important for the successful ingrowth of the implant in the dental bed. In contrast, the colonization of bacteria and their biofilm is a significant problem, leading to peri-implantitis. The weak point in this problem is the interface between the abutment, implant, and soft tissue (the area of the gingival sulcus). Created with BioRender.com.

The attachment of the hard and soft tissue is an essential factor for the successful integration of dental implants. A significant problem when aiming for the successful ingrowth of the implant is the inflammation of the dental bed, known as peri-implantitis. Peri-implantitis is typically triggered by a biofilm formation of periodontal pathogenic bacteria, leading to insufficient healing or bone resorption (osteolysis) of the alveolar bone, which can result in the loss of the implant. In particular, the interface of the abutment, implant, and soft tissue, more precisely the area of the gingival sulcus, is predestinated for the adhesion of bacteria (Figure 1) [1,2,3]. For this reason, it is important to support the cell growth of soft tissue and bone around the dental implant and minimize the colonization of bacteria on the implant surface. Therefore, different approaches to surface treatment have been developed to generate antibacterial surfaces, either through chemical antibacterial coatings or by creating hydrophobicity through surface roughness [4,5,6].

Another important factor is the choice of implant material to minimize inflammation. Titanium is the most common material used for implants, but ceramics are being used more and more frequently for this application. In particular, zirconia is a widely used ceramic in dental applications [7,8,9,10]. This is due to the good biocompatibility and the better antibacterial properties of ceramics compared to titanium [11,12,13]. Of particular interest is the use of glass solder for ceramic dental implants, as it is already used to connect the implant components. As a mix of mainly zirconia and aluminum oxide, it features a low softening point, which is important when bonding the individual components. In this context, the low softening point could also be a disadvantage regarding further surface treatment, as the material is sensitive to heat influence during surface treatment. Due to the common application of spray coating and thermal post-processing, which leads to a smooth surface, glass solder is initially unsuitable as a base material for desired cell growth [14,15].

In addition to bacterial adhesion, the effort required by the cells to grow on the implant is a critical factor. Improved tissue growth behavior due to a certain surface roughness (Ra) of 1–2 µm is already used for dental implants [16,17,18]. Overall, current scientific and industrial efforts aim to create a multifunctional surface with antibacterial properties and surface properties beneficial to cell attachment, which can be achieved by controlling the surface roughness [6,16,19].

Several options for customizing surface roughness are available, ranging from sandblasting to etching [20,21]. However, those conventional surface modifications face process-related limitations in controlling the surface topography. These restrictions can be overcome by ultra-short-pulsed-laser (USPL) processing. USPL offers a precise and nearly athermal method to form a variety of desired surface designs, even on challenging materials, for example, glass or ceramics [22,23,24,25]. Surface designs generated by USPL can be divided into two surface types: laser-inscribed and self-organized structures. Laser-inscribed structures are deterministic patterns corresponding to the laser spot size [26,27]. Delgado-Ruíz et al. showed that it is possible to apply such structures to dental implants made of zirconia featuring a desired surface roughness [28]. Self-organized structures are commonly much smaller than the spot size, and can be indirectly influenced by the laser and the scanning parameters [29]. These parameters include the pulse overlap (*PO*) and line overlap (*LO*). Both scanning strategies have a significant impact, particularly on the formation of self-organized structures [30]. Moreover, the laser process not only changes the surface’s topography and morphology, but also the chemical and crystallographic properties [28,31,32]. This can cause changes in the wettability of the surface [32,33,34]. All these effects can be used to change the properties of the surface implants positively [19,35]. Overall, USPL presents a versatile method for surface functionalization of dental implants, particularly for creating antibacterial surfaces and cell adhesion on sensitive materials such as glass solder.

However, there is no systematic study of the effect of USPL and scanning parameters on a glass solder-coated zirconia substrate. This study aims to identify the factors that influence the formation of different surface morphologies with the potential to serve as functional surface structures for dental implants. Therefore, this study is designed to generate self-organized structures on glass solder using a 300 fs laser system. Laser fluence and scanning parameters are varied, to create different surface topographies. Confocal laser scanning microscopy (CLSM) is used to evaluate the surface roughness and ablation depth after USPL processing. Scanning electron microscopy (SEM) is also used to characterize the surface morphology of the resulting surface structure after USPL processing. Based on the results, a parameter window is defined for the use of an infrared laser wavelength.

## 2. Materials and Methods

### 2.1. Material

**Figure 2:**
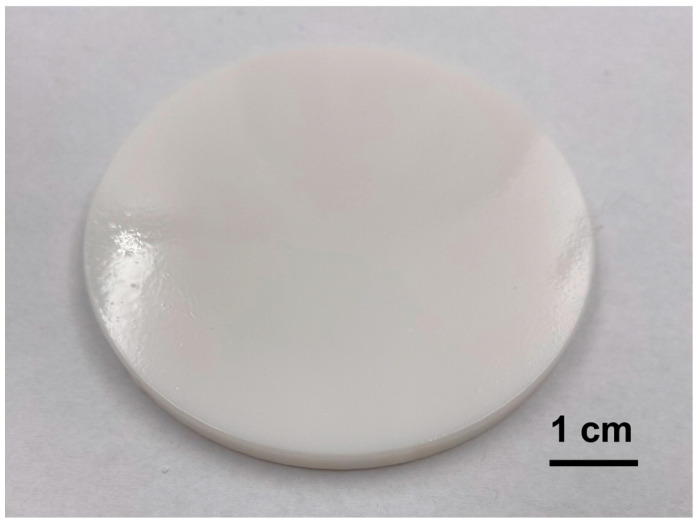
CCD-image of a sample of the glass solder-coated zirconia substrate before laser treatment.

Cylindrical zirconia plates with a 65 mm diameter and 5 mm thickness were used as the base material (Dental Direkt GmbH, Spenge, Germany). Before the plates were coated with a layer of glass solder, they were sandblasted (110 µm Al_2_O_3_, 2 bar) and then cleaned in ethanol in an ultrasonic bath. The main components of the glass solder (DCMhotbond Tizio surface, DCM Dental Creativ Management GmbH, Rostock, Germany) were SiO_2_ (60–62 wt%), Al_2_O_3_ (17–19 wt%), K_2_O (6–8 wt%) and Na_2_O (7–9 wt%). The glass solder is characterized by the following specifications: the coefficient of thermal expansion is 9.4 × 10–6 K^−1^ (25–500 °C), the transformation temperature is 635 °C and the bending strength is ≥50 MPa. The glass solder powder is mixed with a shoulder mass liquid and applied to the cleaned plates, using spin–spray coating. The layer is fired at 1035 °C for 5 min in a vacuum, using a heating and cooling rate of 20 K min^−1^. The coating process was repeated three times, resulting in a final layer thickness of approximately 60 µm. The initial average area roughness of the glass solder surface was determined, using a confocal laser scanning microscope (see Section 2.3), to be 27 ± 1 nm (Figure 2).

### 2.2. Laser Treatment

The structuring process is performed by a femtosecond fiber laser (300 fs, UFFL_60_200_1030_SHG from Active Fiber Systems GmbH, Jena, Germany) integrated into a five-axis micromachining center (Microgantry GU4 from Kugler GmbH, Salem, Germany). The laser system can be operated with an average laser power of up to 60 W and a pulse repetition rate from 50.3 kHz to 18.6 MHz. The emitted wavelength used in this study is 1030 nm and has a linearly polarized beam. The theoretical circular focus diameter of 32 µm at a 1/e^2^ intensity of a Gaussian laser beam profile is created through an f-theta lens with a 163 mm focal length.

The pulse repetition rate was set at 100 kHz during the laser-structuring process. The pulse energy, scanning pulse overlap (*PO*) and scanning line overlap (*LO*) were varied systematically, from 40 to 90%. The variation leads to a geometrically equal pulse energy on the surface and allows the investigation of the influence in terms of time of pulse sequences. The ablation threshold for glass solder was determined, in previous tests, to be a fluence of 2.55 J/cm^2^, which results in a laser pulse energy (*E_P_*) of 20 µJ and is used as the minimum pulse energy applied in this study. The scanning velocity depends on each value of *PO* (Table 1). Pulse and line overlap were calculated according to [30].

Laser scanning was applied to a processing field of 2 mm × 2 mm, using five overscans. Afterward, the samples were cleaned in an ultrasonic bath (Sonorex Super RK100/H, Bandelin electronic GmbH and Co. KG, Berlin, Germany) with ethanol for 10 min, and dried on dust-free wipes (Kimtech Science Precision Wipes, Kimberly-Clark Professional, Dallas, TX, USA).

### 2.3. Surface Characterisation

A confocal laser scanning microscope (CLSM) (LEXT OLS 4000, Olympus, Hamburg, Germany) was used for the analysis of the ablation depth and the average surface roughness (*Sa*). The measurement of the surface roughness and ablation depth was carried out via the integrated software OLS4000 (Version 2.2.3, Olympus, Hamburg, Germany) at an optical magnification of 1080×. The surface roughness was determined without a cut-off wavelength, and each structured field was measured three times. The significance level was set at *p* < 0.05 and the *t*-test was performed using OriginPro 2023 (Version 10.0.0.154, OriginLab Corporation, Northampton, MA, USA). The morphology and periodicity of the generated structures were visualized with a scanning electron microscope (SEM) (SUPRA25 with Gemini-Optical system, Zeiss AG, Oberkochen, Germany). The samples were first coated with a 4 nm thin layer of gold, to ensure conductivity during the SEM process (Sputter-Coater, Emitech Ltd., London, UK). The spatial periodic distance (*λ_d_*) of the created structure was measured with ImageJ (Version 1.54d), using the SEM images.

## 3. Results

### 3.1. Surface Morphology

**Figure 3:**
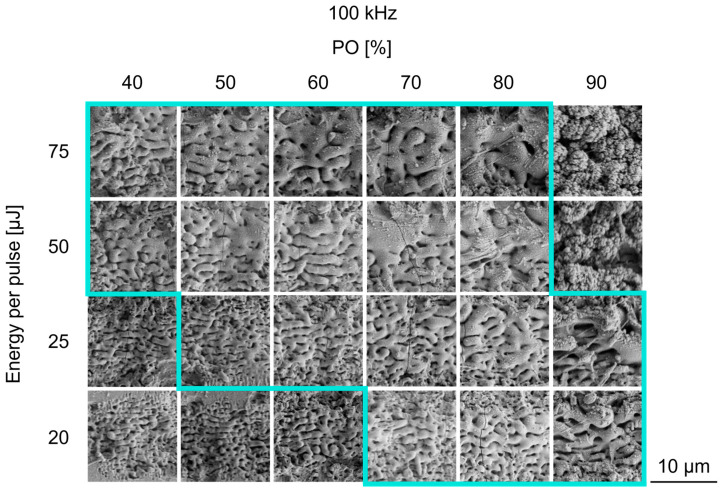
SEM images of the laser-structured glass solder with pulse overlap (*PO*) variation, *LO* = 50%, and different pulse energies, after five total overscans. The process window is marked with a turquoise frame. Outside this process window, there is no homogenous, closed structural pattern (**bottom left**) or excessive ablation (**top right**).

**Figure 4:**
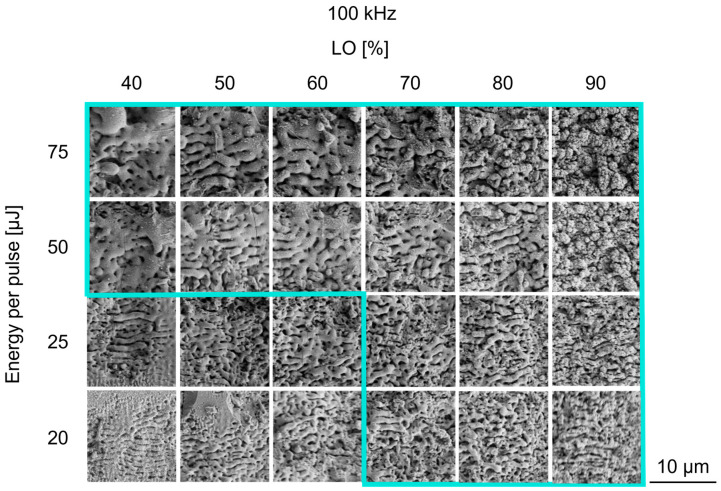
SEM images of the laser-structured glass solder with line overlap (*LO*) variation, *PO* = 50%, and different pulse energies, after five total overscans. The process window is marked with a turquoise frame. Outside this process window, there is no homogenous structure (**bottom left**).

The laser and scanning parameter variations of *PO* and *LO* were successfully applied to the glass solder, as can be seen by the formation of diverse surface morphologies represented in Figure 3 and Figure 4 by SEM images. In the following, the results of the *PO* variation are considered first.

**Figure A1:**
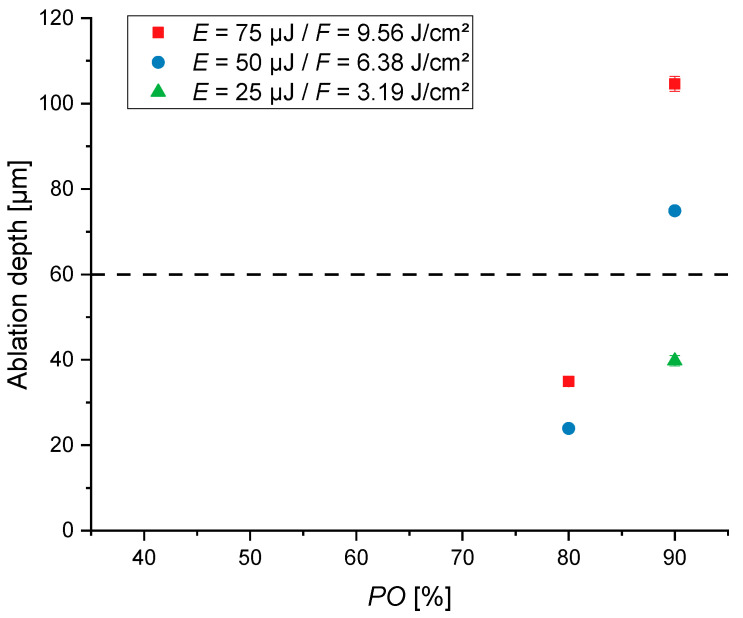
Selected ablation depths with varying *PO* settings. The dashed line marks the layer thickness of the glass solder. Each structured field was measured three times, and all results are statistically significant (*p* < 0.05).

Figure 3 displays an overview of self-organized surface structures achieved by USPL processing, with varying *PO* at different fluences. A parameter window can be determined (marked with a blue frame in Figure 3), which excludes both inhomogeneous coverage of surface structures, as well as excessive ablation depth. At the lowest pulse energy (*E_P_* = 20 µJ) and a relatively low level of *PO*, the SEM images reveal no homogeneous structure on the surface (Figure 3, outside the blue line, bottom left). In contrast, a homogenous structure is formed at the same pulse energy, starting from a *PO* = 70%. Furthermore, measurements of the ablation depth (shown in Figure A1) using CLSM show an increase in the amount of removed material when the pulse energy and *PO* increase. Since the glass solder layer has a thickness of around 60 µm, a maximum ablation depth must be defined to avoid a disappearing glass solder layer and damaging the coating substrate. Thus, the parameter window for using an infrared wavelength can be defined as a pulse energy ranging from 20 to 75 µJ and a pulse overlap from 40 to 90%. Based on these criteria, parameter combinations with no homogeneous structures or excessive ablation depth are excluded from further evaluation in this study.

What generally stands out is the fact that two different structure morphologies can be observed in the SEM images. As can be seen from Figure 3, periodic wave-like structures are formed at low pulse energies up to 25 µJ. These wave-like structures are most prominent at a high *PO* of 90%. Periodic structures with smaller dimensions can also be observed to form at lower levels of POs. It can also be noted that the spatial periodic distance (*λ_d_*) between these waves increases, especially with increasing levels of *PO*: *λ_d_* = 0.81 ± 0.08 µm at *E_P_* = 25 µJ, *PO* = 50% compared to *λ_d_* = 1.40 ± 0.09 µm at *E_P_* = 25 µJ, *PO* = 90%. It can be seen even with the increase of the pulse energy: *λ_d_* = 0.86 ± 0.05 µm at *E_P_* = 20 µJ, *PO* = 70% compared to *λ_d_* = 2.05 ± 0.19 µm at *E_P_* = 75 µJ, *PO* = 70%. The surface morphology is modified to chaotic formations with fused waves when the pulse energy is further raised. Interestingly, the size of those structures’ morphological features (e.g., ridge width) is significantly increased, due to a fusion of single protrusions. This behavior can be observed with the simultaneous increase in *PO* and pulse energy. Furthermore, those surface structures are characterized by the distinct formation of microcracks, which occur at every pulse energy used.

In comparison, Figure 4 shows the SEM images of the variation of the line overlap (*LO*). Similar to the variation of the *PO*, a parameter window can be defined (marked with a blue frame). Structures without homogeneous, closed patterns (bottom left) can also be observed. One of the most striking differences between these SEM images and Figure 3 is the morphology of the structures at low energy pulses. The wave-like structures occur at pulse energies up to 25 µJ and simultaneous *LO* at 70%, but they differ from the previous observations (Figure 3) in their morphology and periodic distance. The spatial periodic distance appears smaller than already observed before: *λ_d_* = 0.69 ± 0.03 µm at *E_P_* = 25 µJ, *LO* = 70% and *λ_d_* = 0.53 ± 0.05 µm at *E_P_* = 25 µJ, *LO* = 90%. But, as observed before, the spatial period distance becomes longer with the increase in the pulse energy: *λ_d_* = 0.69 ± 0.03 µm at *E_P_* = 20 µJ, *LO* = 70%, compared to *λ_d_* = 1.74 ± 0.12 µm at *E_P_* = 75 µJ, *PO* = 70%. With *LO* = 90%, the wave-like structures are overlaid with fine-grained cauliflower-like structures. Interestingly, it can be seen from Figure 4 that with 80–90% *LO*, cauliflower-like structures are formed with the highest pulse energy. Compared to the variation in *PO* (Figure 3), these structures are within the parameter window, as there is no excessive ablation and, in general, smaller grain sizes appear. Their grain size increases simultaneously with the pulse energy from fine-grained to coarse-grained structures. Furthermore, melting occurs at the pulse energy from 50 µJ and an *LO* between 40 and 70%. Microcracks also occur at pulse energies from *E_P_* = 50 µJ.

In sum, the variation of the pulse energy and both scan parameters (*PO* and *LO*) led to the successful structuring of the glass solder-coated zirconia substrate and different surface morphologies. Two structures appeared when varying the *PO* and *LO*: periodic wave-like structures at low pulse energies and cauliflower-like structures at higher pulse energies. When changing the line overlap (*LO*) (Figure 4), cauliflower-like structures start to overlay the wave-like structure. Interestingly, the size and periodicity of the observed periodical structure type changed with the applied laser and scanning parameters. To quantify this observation, roughness measurements are evaluated below.

### 3.2. Surface Topography

**Figure 5:**
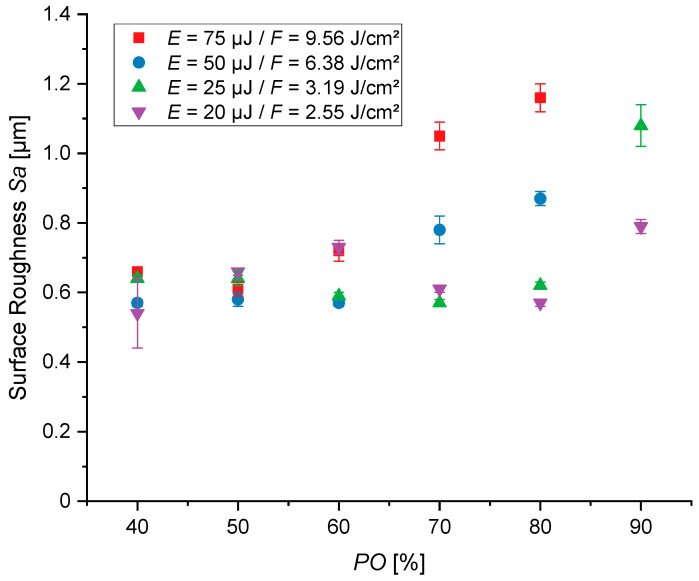
Area surface roughness (*Sa*) with a variation of the pulse overlap (*PO*), energy per pulse (*E*) or average fluence (*F*) and scanning line overlap (*LO*) at 50%, for five overscans. Each structured field was measured three times, and all results are statistically significant (*p* < 0.05).

**Figure 6:**
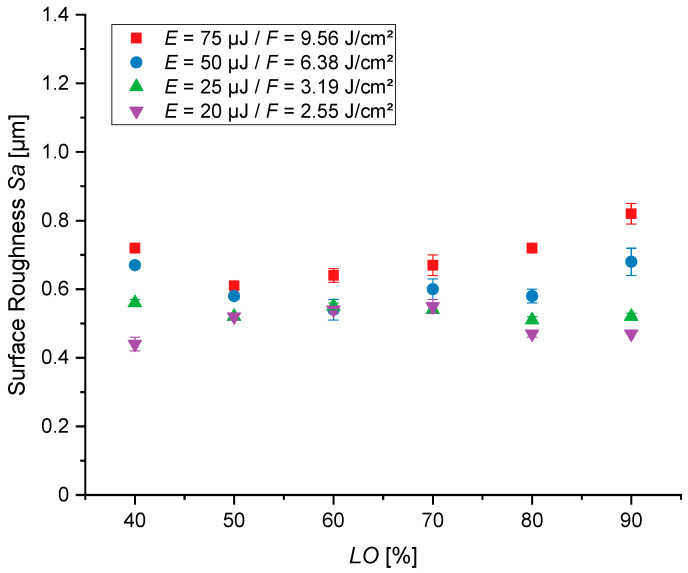
Area surface roughness (*Sa*) with a variation of the line overlap (*LO*), energy per pulse (*E*) or average fluence (*F*) and scanning pulse overlap (*PO*) at 50%, for five over cans. Each structured field was measured three times, and all results are statistically significant (*p* < 0.05).

In addition to the SEM images, the average area surface roughness (*Sa*) of the structures defined in the process window was measured via CLSM (Figure 5 and Figure 6). The measured values of the area surface roughness of the variation of *PO* are shown in Figure 5. The minimum value of the area surface roughness measured is 0.54 ± 0.10 µm at *PO* = 40% at a pulse energy of *E_P_* = 20 µJ, and the maximum value is 1.16 ± 0.04 µm at *E_P_* = 75 µJ with *PO* = 80%. The roughness achieved at the lowest energy per pulse barely changes, and is in the range of *Sa* = 0.54 ± 0.10 to 0.79 ± 0.02 µm, with varying *PO* settings. At the other pulse energies, the roughness increases almost equally with the increase in *PO*. The trend graphs of the roughness data have their highest positive gradient at both the highest pulse energies with a *PO* of 70%. This is in line with the merge of single protrusions observed in Figure 2.

Figure 6 shows the measured area surface roughness for the variation of the *LO* at different pulse energies. The minimum area surface roughness of *Sa* = 0.44 ± 0.02 µm is achieved with an *LO* of 40% at a pulse energy of 20 µJ. The highest observed roughness of 0.82 ± 0.03 µm occurs at a pulse energy of 75 µJ and *LO* = 90%. What stands out in this figure is the generally smaller value range in which all measured roughness values are located, compared to the variation of *PO*. There is no drastic gradient of the roughness with the variation of *LO*, when compared to Figure 5. Even the appearance of cauliflower-like structures has no strong increase in the roughness. Overall, the roughness values are lower than in Figure 5.

On the one hand, relating the different roughness values by changing the laser and scanning parameters shows that the surface topography can be finely adjusted, from 0.44 ± 0.02 µm up to 1.16 ± 0.04 µm in the defined parameter window. On the other hand, these graph trends of Figure 5 and Figure 6 reveal that different rises occur when comparing the *PO* and *LO* variations. Using an increased *PO*, an exponential-like graph trend stands out. In contrast, when the *LO* is raised, a more linear trend occurs.

## 4. Discussion

This study investigated the effects of varying laser parameters (*PO* and *LO*) on a glass solder-coated zirconia substrate. The aim was to achieve a geometrically equal pulse energy on the surface by varying *PO* and *LO*. This allows the investigation of the influence in terms of time of pulse sequences. Different conclusions can be drawn, considering the morphological and topographical analysis results of the structured glass solder surface. We showed that the applied pulse overlap and the line overlap significantly affected the type and periodicity of the resulting surface structures.

The initial step was to define a process window that excluded inhomogeneous distributions of surface structures and ensured that the ablation depth did not exceed the thickness of the glass solder coating. The inhomogeneous cover of the surface structure can be related to the Gaussian laser beam used in context with the ablation threshold of the material. Using a low fluence and a low *PO* or LO, the drop in intensity in the outer area of the Gaussian beam profile leads to an insufficient ablation. Thus, only the center of the focused laser spot leads to ablation. As the fluence increases, the intensity level within the outer regions of the Gaussian beam also rises, resulting in material ablation that occurs throughout the entire focused area. This phenomenon has already been described in several studies [30,36]. When increasing the *PO* or *LO*, the number of applied pulses per area rises and a breakdown of the ablation threshold of the material can be reached. As a result, each pulse damages the underlying material, which can subsequently lead to lowering the necessary ablation fluence. This is explained as the incubation effect, and was reported by Güdde et al. [37].

Furthermore, the applicable laser and scanning parameters are restricted by an ablation depth exceeding the coating thickness of the glass solder layer. This case occurs especially when using a *PO* of 90% and relatively high pulse energies. This indicates a strong heat accumulation in the glass solder, due to the high number of laser pulses in the same area on the material [38]. This results in excessive ablation of the glass solder layer and the zirconia base material being reached. With a reduction in the layer thickness of the glass solder, the process window would become smaller. Overall, these results confirm the importance of optimizing the process parameters to achieve structuring for roughness modification of the glass solder without undesired side effects.

In the parameter window, the results show that two surface morphologies can be generated. Using the variation of the *PO*, primarily wave-like structures are formed. These wave-like structures feature an Sa of 0.54 ± 0.10 µm to 1.16 ± 0.04 µm and a preferential orientation parallel to the scanning direction. Their spatial periodic distance varies from 0.81 ± 0.08 µm to 2.05 ± 0.19 µm. When considering the morphology of the wave-like structures closely, dominant signs of melt formation are visible. Thus, it can be assumed that heat-driven phenomena are the mechanism responsible for this structure type. At high pulse repetition rates (e.g., several hundred kHz) and high POs, as used in this study, heat accumulation during processing becomes crucial, as there is not enough time between the pulses for the dissipation of introduced heat [39]. During the laser-structuring process, the surface temperature can be deliberately increased up to the softening point, which can change the state of the material [28,38,40]. This interplay between the applied pulse repetition rate, pulse overlap, and used pulse energy significantly affected the ablation and surface structure formation on the glass solder, even at a relatively low repetition rate of 100 kHz, used in this study due to the low transformation temperature (635 °C). The significant role of heat accumulation is further confirmed by cracks at the same point where wave-like structures are formed. Further studies using X-ray diffraction analysis (XRD) are necessary to reveal crystallographic phase changes inside the near-surface layer and validate the assumption of the predominantly heat-driven formation of wave-like structures on the glass solder.

With the variation of *LO*, the wave-like structures also appear. However, these are overlaid by fine-grained cauliflower-like structures at low pulse energies and high *LO*. The cauliflower-like structures are mainly prominent at high *LO* and high pulse energy. The measured surface roughness of these structures ranges from 0.44 ± 0.02 µm to 0.82 ± 0.03 µm. In contrast to the waves, the structure is characterized by a chaotic orientation with a spatial periodic distance between 0.53 ± 0.05 µm and 1.74 ± 0.12 µm. Based on these results, *LO* has a lower influence on heat accumulation than *PO*, as less melting occurs. The time difference between applied laser pulses by subsequent scanning lines during laser treatment is, therefore, long enough so that heat accumulation does not become excessive.

In general, the investigations of the surface roughness using CLSM showed that the area roughness of the structures varied, depending on the applied laser and scanning parameters. In summary, the study indicates that the variation of *PO* and *LO* significantly influences the morphology and roughness of the structured glass solder surfaces. Both parameters produce different types of structure, and consequently different surface roughness values, whereby excessive ablation and melt formation can cause undesirable effects. The *PO* has a slightly stronger influence on the characteristics of the wave-like structure, and higher roughness values are achieved. The specified parameter windows enable the generation of homogeneous structures with controllable surface roughness. For a potential application on a dental implant, the average area surface roughness values must be considered. The literature compares the average area surface roughness of several dental implants. The average area surface roughness of the most common zirconia implants is between 0.2 and 2 µm [41]. The roughness values achieved in our study are within this range. Increasing the values of the area surface roughness on glass solder would be conceivable, by creating more hierarchical structures using a combination of laser-structured trenches and the created wave-like and cauliflower-like structures. However, in the absence of empirical data concerning cell and bacterial adhesion to the structures created in this study, the results of cell experiments must be awaited before further optimization of the structural design. The influence of laser structuring on the adhesion of the glass solder to the zirconia must also be investigated. This has only been investigated in unstructured samples [42].

Our results are partly consistent with those of Xu et al. and Höhm et al., which describe similar effects of laser processing of fused silica and silica under different laser wavelengths and parameters [43,44]. Xu et al. describe the appearance of fluffy structures and laser-induced periodic surface structures on fused silica [43]. The fluffy structures are morphologically comparable to the cauliflower-like structures in this study, occurring at high scan speed and resulting in high *PO*. In the study of Höhm et al., various nanostructures were formed, and so-called LIPSS also occurred on silica [44]. These laser-induced periodic surface structures are caused by interference effects between the laser light and the material [45]. This is explained by surface electromagnetic waves or surface plasmon polaritons, resulting in the characteristic waveform [46]. In addition, variations of LIPSS can be observed on various glass types [33]. The morphology of the occurring wave-like structures is similar to the LIPSS on glasses in previous studies. The occurrence of chaotic formations with the increase of the pulse energy is also observed by Kunz et al. [47]. In the study by Xu et al., “the period [of the LIPSS] increased with the increase of the laser fluence” [43]. As described above, we are able to make the same observations. The spatial periodic distances we observe and the parallel orientation of the wave-like structures to the polarization of the laser beam are comparable to the values observed by Höhm et al. and Gräf et al. [33,44]. The wave-like structures can, therefore, be categorized as slow spatial-frequency LIPSS (LSFL). Furthermore, the combination of selected wavelength and material influences the expression of LIPSS, which has been shown in studies on different ceramics and glasses. Moreover, different light–material interactions on the top glass-solder layer are present, compared to well-known metal or glass substrates, resulting in new physical and chemical surface properties. The choice of the wavelength influences the generation of LIPSS, especially with challenging materials such as glass [33,47,48,49].

## 5. Conclusions

This study shows that a glass solder zirconia double-layer substrate can be successfully structured by using a femtosecond laser. The glass solder zirconia double-layer substrate offers a new challenge in femtosecond laser processing, since processing parameters must be finely adjusted to keep the bonding of both materials unaltered. Two different structure morphologies can be created: wave-like structures, more specifically, low-spatial-frequency LIPSS (LSFL), and cauliflower-like structures. The characteristics of these structures depend on the choice of the laser parameters, like wavelength and pulse energy. However, as shown in this study, another important factor is also the pulse overlap, as a laser scanning parameter. Further investigations will address the wetting, mechanical, and biological properties, such as cell experiments of both surface morphologies generated by femtosecond laser treatment.

## Figures and Tables

**Table 1:** The laser scanning velocity (*v*) is selected, depending on the pulse overlap (*PO*).

*PO* [%]	*v* [m/s]
40	1.9
50	1.6
60	1.3
70	0.9
80	0.7
90	0.3

**Figure A2:**
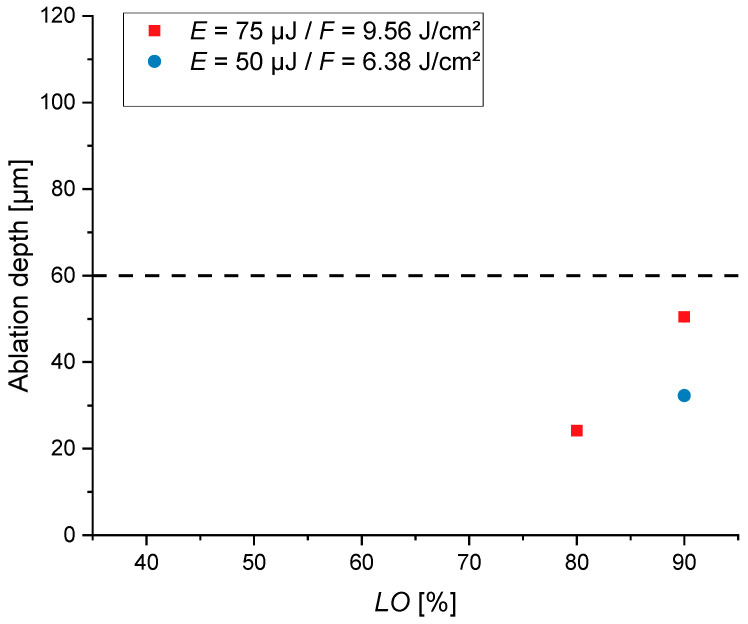
Selected ablation depths with varying *LO* settings. The dashed line marks the layer thickness of the glass solder. Each structured field was measured three times, and all results are statistically significant (*p* < 0.05).

## Data Availability

The data presented in this study are available on request from the corresponding author. The data are not publicly available, due to privacy.
